# Supplementary material for: Real-time closed-loop feedback system for mouse mesoscale cortical signal and movement control
Source: eLife. 2026 Sep 24;14:RP105070. doi: 10.7554/eLife.105070 (PMC13609482; doi:10.7554/eLife.105070)
Supplement: Peer review history

# Editor report: eLife Assessment

**DOI:** 10.7554/eLife.105070.4.sa0

**Authors:** Juan Alvaro Gallego

**Affiliations:** https://ror.org/03g001n57 Champalimaud Foundation Portugal

**Keywords:** Convincing, Valuable

This study presents a platform to implement closed-loop experiments in mice based on auditory feedback. The authors provide **convincing** evidence that their platform enables a variety of closed-loop experiments using neural or movement signals, indicating that it will be a **valuable** resource to the neuroscience community. The authors make this platform more accessible by complementing the paper with a detailed tutorial explaining how to implement the software and hardware.

# Referee report: Reviewer #1 (Public review)

**DOI:** 10.7554/eLife.105070.4.sa1

[Editors' note: this version has been assessed by the Reviewing Editor without further input from the original reviewers. The authors have addressed the comments raised in the previous round of review.]

Summary:

The authors provide a resource to the systems neuroscience community by offering their Python-based CLoPy platform for closed-loop feedback training. In addition to using neural feedback, as is common in these experiments, they include a capability to use real-time movement extracted from DeepLabCut as the control signal. The methods and repository are detailed for those who wish to use this resource. Furthermore, they demonstrate the efficacy of their system through a series of mesoscale calcium imaging experiments. These experiments use a large number of cortical regions for the control signal in the neural feedback setup, while the movement feedback experiments are analyzed more extensively. The revised preprint has improved substantially upon the previous submission.

Strengths:

The primary strength of the paper is the availability of their CLoPy platform. Currently, most closed-loop operant conditioning experiments are custom built by each lab, and carry a relatively large startup cost to get running. This platform lowers the barrier to entry for closed-loop operant conditioning experiments, in addition to making the experiments more accessible to those with less technical expertise.

Another strength of the paper is the use of many different cortical regions as control signals for the neurofeedback experiments. Rodent operant conditioning experiments typically record from the motor cortex, and maybe one other region. Here, the authors demonstrate that mice can volitionally control many different cortical regions not limited to those previously studied, recording across many regions in the same experiment. This demonstrates the relative flexibility of modulating neural dynamics, including in non-motor regions.

Finally, adapting the closed-loop platform to use real-time movement as a control signal is a nice addition. Incorporating movement kinematics into operant conditioning experiments has been a challenge due to the increased technical difficulties of extracting real-time kinematic data from video data at a latency where it can be used as a control signal for operant conditioning. In this paper, they demonstrate that the mice can learn the task using their forelimb position, at a rate that is quicker than the neurofeedback experiments.

# Referee report: Reviewer #2 (Public review)

**DOI:** 10.7554/eLife.105070.4.sa2

Summary:

In this work, Gupta & Murphy present several parallel efforts. On one side, they present the hardware and software they use to build a head-fixed mouse experimental setup that they use to track in "real-time" the calcium activity in one or two spots at the surface of the cortex. On the other side, they present another setup that they use to take advantage of the "real-time" version of DeepLabCut with their mice. The hardware and software that they used/develop is described at length, both in the article and in a companion GitHub repository. Next, they present experimental work that they have done with these two setups, training mice to max out a virtual cursor to obtain a reward, by taking advantage of auditory tone feedback that is provided to the mice as they modulate either (1) their local cortical calcium activity, or (2) their limb position.

Strengths:

This work illustrates the fact that thanks to readily available experimental building blocks, body movement and calcium imaging can be carried out using readily available components, including imaging the brain using an incredibly cheap consumer electronics RGB camera (RGB Raspberry Pi Camera). It is a useful source of information for researchers that may be interested in building a similar setup, given the highly detailed overview of the system. Finally, it further confirms previous findings regarding the operant conditioning of the calcium dynamics at the surface of the cortex (Clancy et al. 2020) and suggests an alternative based on deeplabcut to the motor tasks that aim to image the brain at the mesoscale during forelimb movements (Quarta et al. 2022).

# Referee report: Reviewer #3 (Public review)

**DOI:** 10.7554/eLife.105070.4.sa3

The study demonstrates the effectiveness of a cost-effective closed-loop feedback system for modulating brain activity and behavior in head-fixed mice. Authors have tested real-time closed-loop feedback system in head-fixed mice two types of graded feedback: (1) Closed-loop neurofeedback (CLNF), where feedback is derived from neuronal activity (calcium imaging), and (2) Closed-loop movement feedback (CLMF), where feedback is based on observed body movement. It is a python based opensource system, and the authors call it CLoPy. Authors also claim to provide all software, hardware schematics, and protocols to adapt it to various experimental scenarios. This system is capable and can be adapted for a wide use case scenarios.

Authors have shown that their system can control both positive (water drop) and negative reinforcement (buzzer-vibrator). This study also shows that using the closed-loop system, mice have shown to better performance, learnt arbitrary tasks and can adapt to changes in the rules as well. By integrating real-time feedback based on cortical GCaMP imaging and behavior tracking authors have provided strong evidence that such closed-loop systems can be instrumental in exploring the dynamic interplay between brain activity and behavior.

# Author comment: Author response

**DOI:** 10.7554/eLife.105070.4.sa4

**Authors:** Pankaj Kumar Gupta, Timothy H Murphy

**Affiliations:** https://ror.org/03rmrcq20 University of British Columbia Vancouver Canada; https://ror.org/03rmrcq20 University of British Columbia Vancouver Canada

The following is the authors’ response to the previous reviews

**Public Reviews:**

**Reviewer #1 (Public review):**

Summary:

The authors provide a resource to the systems neuroscience community by offering their Python-based CLoPy platform for closed-loop feedback training. In addition to using neural feedback, as is common in these experiments, they include a capability to use real-time movement extracted from DeepLabCut as the control signal. The methods and repository are detailed for those who wish to use this resource. Furthermore, they demonstrate the efficacy of their system through a series of mesoscale calcium imaging experiments. These experiments use a large number of cortical regions for the control signal in the neural feedback setup, while the movement feedback experiments are analyzed more extensively. The revised preprint has improved substantially upon the previous submission.

Strengths:

The primary strength of the paper is the availability of their CLoPy platform. Currently, most closed-loop operant conditioning experiments are custom built by each lab, and carry a relatively large startup cost to get running. This platform lowers the barrier to entry for closed-loop operant conditioning experiments, in addition to making the experiments more accessible to those with less technical expertise.

Another strength of the paper is the use of many different cortical regions as control signals for the neurofeedback experiments. Rodent operant conditioning experiments typically record from the motor cortex, and maybe one other region. Here, the authors demonstrate that mice can volitionally control many different cortical regions not limited to those previously studied, recording across many regions in the same experiment. This demonstrates the relative flexibility of modulating neural dynamics, including in non-motor regions.

Finally, adapting the closed-loop platform to use real-time movement as a control signal is a nice addition. Incorporating movement kinematics into operant conditioning experiments has been a challenge due to the increased technical difficulties of extracting real-time kinematic data from video data at a latency where it can be used as a control signal for operant conditioning. In this paper, they demonstrate that the mice can learn the task using their forelimb position, at a rate that is quicker than the neurofeedback experiments.

Weaknesses:

Many of the original weaknesses have been addressed in the revised preprint.

While the dataset contains an impressive amount of animals and cortical regions for the neurofeedback experiment, my excitement for these experiments is tempered by the relative incompleteness of the dataset.

As we have responded earlier, we acknowledge that some of the neurofeedback experiments include data from only a single mouse for some cortical regions, while for some cortical regions, there are several animals. This was due to practical constraints during the study, and we understand the limitations this poses for drawing broad conclusions. We felt it was still important to include these data sets with smaller sample sizes, as they might be useful for others pursuing this direction in the future. To address this, we have revised the text to explicitly acknowledge these limitations and clarify that the results for some regions are exploratory in nature. We believe our flexible tool will provide a means for our lab and others to include more animals representing additional cortical regions in future studies. Importantly, we have included all raw and processed data as well as code for future analysis.

Additionally, adoption of the platform may be hindered by the absence of a tutorial on how to run a session.

We thank the reviewer for this valuable suggestion. We agree that the absence of clear documentation and tutorials could limit the accessibility and broader adoption of the platform. In response, we have significantly improved the available resources by adding a comprehensive tutorial. Specifically, we have created a dedicated “Wiki” section on the GitHub repository, along with detailed documentation hosted on ReadTheDocs (https://clopy-docs.readthedocs.io). These resources now provide step-by-step guidance on setting up and running a session, along with additional usage examples to facilitate ease of use for new users.

**Reviewer #2 (Public review):**

Summary:

In this work, Gupta & Murphy present several parallel efforts. On one side, they present the hardware and software they use to build a head-fixed mouse experimental setup that they use to track in "real-time" the calcium activity in one or two spots at the surface of the cortex. On the other side, they present another setup that they use to take advantage of the "real-time" version of DeepLabCut with their mice. The hardware and software that they used/develop is described at length, both in the article and in a companion GitHub repository. Next, they present experimental work that they have done with these two setups, training mice to max out a virtual cursor to obtain a reward, by taking advantage of auditory tone feedback that is provided to the mice as they modulate either (1) their local cortical calcium activity, or (2) their limb position.

Strengths:

This work illustrates the fact that thanks to readily available experimental building blocks, body movement and calcium imaging can be carried out using readily available components, including imaging the brain using an incredibly cheap consumer electronics RGB camera (RGB Raspberry Pi Camera). It is a useful source of information for researchers that may be interested in building a similar setup, given the highly detailed overview of the system. Finally, it further confirms previous findings regarding the operant conditioning of the calcium dynamics at the surface of the cortex (Clancy et al. 2020) and suggests an alternative based on deeplabcut to the motor tasks that aim to image the brain at the mesoscale during forelimb movements (Quarta et al. 2022).

Weaknesses:

This work covers 3 separate research endeavors: (1) The development of two separate setups, their corresponding software. (2) A study that is highly inspired from the Clancy et al. 2021 paper on the modulation of the local cortical activity measured through a mesoscale calcium imaging setup. (3) A study of the mesoscale dynamics of the cortex during forelimb movements learning. Sadly, the analyses of the physiological data appears incomplete, and more generally, the paper shows weaknesses regarding several points:

The behavioral setups that are presented are representative of the state of the art in the field of mesoscale imaging/head fixed behavior community, rather than a highly innovative design. Still, they definitely have value as a starting point for laboratories interested in implementing such approaches.

We agree with the reviewer that the behavioral setup presented here reflects current state-of-the-art approaches in the mesoscale imaging and head-fixed behavior community, and that similar systems have been implemented in other laboratories. However, the primary contribution of our work lies not in introducing a fundamentally new design but in providing a fully open-source, modular, and accessible implementation of such a system. By detailing both the hardware and software components, along with protocols for assembly and use, we aim to lower the barrier to entry for laboratories that may lack the specialized expertise or resources required to develop these systems independently. We hope this accessibility and ease of adoption will facilitate broader use of closed-loop and mesoscale imaging approaches across the field.

Throughout the paper, there are several statements that point out how important it is to carry out this work in a closed-loop setting with an auditory feedback. Still, sadly there is no "no feedback" control in cortical conditioning experiments. At the same time, there is a no-feedback condition in the forelimb movement study, which shows that learning of the task can be achieved in the absence of feedback.

We appreciate the reviewer’s insightful comment. We acknowledge that a no-feedback control group was not included in the neurofeedback experiments. This was due in part to the extensive exploration of multiple ROI combinations, as well as preliminary pilot experiments with a no-feedback condition that did not show consistent evidence of learning. Based on these initial results, we chose to prioritize conditions with feedback and did not pursue the no-feedback experiments further. We agree that including such a control would strengthen the study and consider this an important direction for future work.

The analysis of the closed-loop neuronal data behavior lacks controls. Increased performance can be achieved by modulating actively only one of the two ROIs, this is not really analyzed, while this finding which does not match previous reports (Clancy et al. 2020) would be important to further examine.

We agree that further analysis of this aspect would strengthen the interpretation of the dataset, and we encourage the community to explore this question using the publicly released data. In our 2-ROI paradigm, we observed that mice often adopt a strategy of predominantly modulating a single ROI to achieve task success, rather than dynamically balancing both regions. This behavior is noted in the manuscript. Importantly, our task design did not impose explicit constraints on the directionality of modulation across ROIs (i.e., increasing one while decreasing the other), in contrast to the paradigm used in Clancy et al. (2020). This difference in task structure may account for the observed divergence in strategies and outcomes.

**Reviewer #3 (Public review):**

Summary:

The study demonstrates the effectiveness of a cost-effective closed-loop feedback system for modulating brain activity and behavior in head-fixed mice. Authors have tested real-time closed-loop feedback system in head-fixed mice two types of graded feedback: (1) Closed-loop neurofeedback (CLNF), where feedback is derived from neuronal activity (calcium imaging), and (2) Closed-loop movement feedback (CLMF), where feedback is based on observed body movement. It is a python based opensource system, and the authors call it CLoPy. Authors also claim to provide all software, hardware schematics, and protocols to adapt it to various experimental scenarios. This system is capable and can be adapted for a wide use case scenarios.

Authors have shown that their system can control both positive (water drop) and negative reinforcement (buzzer-vibrator). This study also shows that using the closed-loop system, mice have shown to better performance, learnt arbitrary tasks and can adapt to changes in the rules as well. By integrating real-time feedback based on cortical GCaMP imaging and behavior tracking authors have provided strong evidence that such closed-loop systems can be instrumental in exploring the dynamic interplay between brain activity and behavior.

Strengths:

Simplicity of feedback systems design. Simplicity of implementation and potential adoption.

Weaknesses:

Long latencies, due to slow Ca2+ dynamics and slow imaging (15 FPS), may limit the application of the system.

We agree that the latency introduced by calcium dynamics and imaging frame rates is an inherent limitation of calcium imaging–based approaches. Future improvements, including faster calcium indicators, higher frame-rate imaging systems, and more efficient computational pipelines, are expected to mitigate these constraints and enhance temporal precision.

**Recommendations for the authors:**

**Reviewer #1 (Recommendations for the authors):**

This version is a substantial improvement from the previous version. My main recommendation is to add a tutorial, with visualizations of some sort, to show how to run a session with the platform. The tutorials for the probe trajectory planner PinPoint is a good example for reference (https://virtualbrainlab.org/pinpoint/tutorial.html).

We thank the reviewer for this valuable suggestion. We agree that the absence of clear documentation and tutorials could limit the accessibility and broader adoption of the platform. In response, we have significantly improved the available resources by adding a comprehensive tutorial. Specifically, we have created a dedicated “Wiki” section on the GitHub repository, along with detailed documentation hosted on ReadTheDocs (https://clopy-docs.readthedocs.io). These resources now provide step-by-step guidance on setting up and running a session, along with additional usage examples to facilitate ease of use for new users.
